# Quantitative Evaluation of Respiratory Dysfunction and Its Relationships With Parkinson’s Disease Symptoms

**DOI:** 10.1155/padi/9026870

**Published:** 2026-10-13

**Authors:** A. A. Pilipovich, O. V. Vorob’eva, S. A. Makarov, N. N. Shindryaeva

**Affiliations:** ^1^ Department of Nervous Diseases Sechenov First Moscow State Medical University Moscow Russia mma.ru; ^2^ City Polyclinic No. 2 Moscow Health Department Moscow Russia

**Keywords:** dopaminergic therapy, fatigue, nonmotor symptoms, Parkinson’s disease, quality of life, respiratory dysfunction, spirometry

## Abstract

Respiratory disorders significantly impair functional baseline and quality of life (QoL) in Parkinson’s disease (PD). This cross‐sectional study aimed to evaluate spirographic lung function parameters in PD and determine their relationships with motor and nonmotor symptoms, QoL, and dopaminergic therapy. Spirometric parameters were recorded in 57 outpatients with PD and 10 healthy controls. PD patients underwent clinical assessments using UPDRS Parts II and III, Schwab and England scale, PDQ‐39, and PFS‐16. Spirometric analysis revealed significant decreases in VC %pred in PD patients versus controls, and in FVC %pred in PD Stage III versus PD Stage II. Decreased lung function correlated with H&Y stage (VC, FVC, FEV_1_) and showed inverse correlations with UPDRS Parts II and III scores, most pronounced for axial‐locomotor impairments (posturing, gait, stability, arising from chair) and bradykinesia. PFS‐16 fatigue index inversely correlated with FEV_1_ and PEF. Worse QoL (PDQ‐39 summary index, emotional well‐being, and cognition domains) was primarily coupled with decreased expiratory flow rates (FEV_1_, PEF). Untreated PD patients exhibited significantly lower FVC%pred, FEV_1_%pred, FEF_75_%pred, and Tiffeneau index than those on dopaminergic therapy. This study demonstrates the presence of respiratory dysfunction in PD Stages II and III and its negative association with QoL. The results highlight the relationship between lung function decline and overall PD progression, with the strongest associations revealing the most significant contribution of postural abnormalities, bradykinesia, and pathological fatigue. Furthermore, the obtained data suggest a potential compensating role of dopaminergic therapy.

## 1. Introduction

Respiratory dysfunctions are of paramount importance in Parkinson’s disease (PD), as they significantly impair functional capabilities and quality of life (QoL) in PD patients, while remaining one of the primary causes of mortality in this patient population [1–3].

To date, a complete understanding of the exact mechanisms underlying respiratory deficits in PD is still lacking [4]. PD‐related respiratory dysfunction is considered to involve both central mechanisms, linked to neuronal loss within brainstem respiratory control centers, and peripheral components directly associated with respiratory mechanics, including the functioning of the airways and respiratory musculature [5, 6]. Peripheral impairments are frequently associated with a hyperkyphotic posture that restricts lung volume, increased chest wall rigidity, and altered respiratory muscle activity. The decline in lung function and respiratory muscle strength has been shown to correlate with the severity of bradykinesia and rigidity [7–9]. It is hypothesized that these hallmark motor symptoms are coupled with a gradual decrease in chest wall elasticity, a reduction in the range of thoracic movement, and progressive muscle weakness [10, 11]. Consequently, airway patency may diminish and tidal volumes decrease, predisposing patients to the development of mixed obstructive and restrictive ventilatory patterns [3, 12].

Ventilatory problems can manifest at the very early stages of the disease [8, 13]. For instance, Kaminsky et al. [14] demonstrated that lung function in PD patients declined over a 1‐year period compared to healthy controls. In some cases, stridor and dyspnea are observed, with the latter potentially associated with a significant decrease in arterial oxygen partial pressure even at rest. These changes may further exacerbate pathological fatigue and impede the performance of daily tasks. Notably, respiratory dysfunction is frequently asymptomatic, which can predispose patients to silent aspiration, atelectasis, and pneumonia, particularly in advanced stages of PD. Even in the absence of overt clinical manifestations of respiratory distress, spirographic parameters in PD patients are often significantly lower than those in healthy controls [11]. A series of studies have demonstrated a reduction in baseline metrics, such as vital capacity (VC), forced vital capacity (FVC), forced expiratory volume (FEV), and maximum voluntary ventilation (MVV), alongside other impairments [15, 16] that warrant further detailed investigation.

Therapeutic options for managing respiratory dysfunction in PD remain markedly limited. Therefore, clarifying the potential impact of dopaminergic therapy on respiratory parameters is a critical task, although current literature on this matter remains highly contradictory. On the one hand, several studies have reported a positive effect of levodopa therapy on respiratory function, including improvements in obstructive and restrictive patterns, upper airway muscle tone [17, 18], and respiratory muscle strength, which is reflected in the normalization of VC, FVC, FEV in 1 s (FEV_1_), and peak expiratory flow (PEF) values [19–22]. The results of a recent systematic review and meta‐analysis from 2021 [23] suggested that obstructive respiratory patterns are relatively sensitive to dopaminergic stimulation, highlighting an increased risk of respiratory function deterioration when such therapy is unavailable. On the other hand, various adverse effects of levodopa on breathing mechanics have been documented, including drug‐induced dyspnea, tachypnea, hyperventilation, respiratory and diaphragmatic dyskinesias, and other central respiratory arrhythmias [24–28]. Consequently, the net effect of dopaminergic stimulation on the respiratory system remains ambiguous and not fully elucidated, leading to conflicting data across published reports [5].

Further in‐depth, exploratory investigations of changes in pulmonary function tests (PFTs) in PD are essential for timely diagnosis and management. Recognizing specific ventilatory abnormalities will help clinicians optimize treatment strategies, potentially reducing the negative impact of respiratory insufficiency on the overall health and survival of PD patients.

The main objective of this study was to evaluate spirographic lung function parameters in outpatients with PD and to determine their relationships with PD motor symptoms, fatigue, QoL, and dopaminergic therapy.

## 2. Materials and Methods

### 2.1. Patient Recruitment and Ethical Approval

Patient recruitment was carried out at the clinical sites of the Department of Nervous Diseases of the Institute of Professional Education at Sechenov University. The study was approved by the Local Ethics Committee of Sechenov University (Protocol No. 04‐13 dated April 10, 2013).

### 2.2. Inclusion and Exclusion Criteria

The inclusion criteria for the study were as follows:

• men and women aged ≥ 18 years;
• a clinically established diagnosis of idiopathic PD in accordance with the United Kingdom Parkinson’s Disease Society Brain Bank clinical diagnostic criteria [29];
• signed voluntary informed consent provided by the patient.

The exclusion criteria were as follows:

• symptomatic (secondary) forms of parkinsonism and “parkinsonism‐plus” syndromes;
• the presence of moderate or severe dementia (according to the ICD‐10 criteria);
• concomitant severe psychiatric, somatic, or neurological pathology that could interfere with the correct performance of the study;
• a history of smoking or smoking at the time of the examination.

The control group included healthy individuals with no clinical signs of PD or concomitant respiratory pathology, who met the same exclusion criteria and were matched with the main group by age and sex.

### 2.3. Clinical and Neurological Assessment

Clinical examinations of all patients were performed by the same investigator during the “ON” period (the medication phase under the effect of the patient’s current dose of levodopa‐containing drugs).

The neurological examination included the following:

• disease staging using the classic Hoehn and Yahr (H&Y) rating scale;
• motor and functional scoring via the Unified Parkinson’s Disease Rating Scale (UPDRS), specifically Part II (activities of daily living) and Part III (motor examination);
• functional independence evaluation using the Schwab and England activities of daily living scale (Sch&En).

QoL was evaluated using the specialized 39‐item Parkinson’s Disease Questionnaire (PDQ‐39) [30]. The severity of fatigue was assessed using the 16‐item Parkinson’s Disease Fatigue Scale (PFS‐16) [31].

### 2.4. PFT

Pulmonary function parameters were evaluated via spirometry using a computerized “CHESTGRAPH HI‐105” spirometer (Chest, Japan). The testing protocol included the registration of the following metrics:

• VC;
• FVC;
• FEV_1_;
• Tiffeneau index (IT) (FEV_1_/VC ratio);
• Gensler index (FEV_1_G) (FEV_1_/FVC ratio);
• PEF;
• maximal midexpiratory flow (MMF);
• forced expiratory flows at 25%, 50%, and 75% of FVC (FEF_25_, FEF_50_, FEF_75_);
• MVV.

All spirometric parameters were recorded in absolute values (liters, liters per second) and automatically converted into percentages of predicted values (% pred) adjusted for each patient’s individual sex, age, height, and weight. Spirometric analysis and data interpretation were performed according to the international standards of the American Thoracic Society (ATS) using the Knudson reference equations, which allow for the correct evaluation of respiratory function in individuals over 70 years of age [32, 33].

### 2.5. Statistical Analysis

Statistical processing of the data was performed using IBM SPSS Statistics software. Due to the non‐normal distribution of variables (as assessed by the Kolmogorov–Smirnov and Shapiro–Wilk tests), continuous data are presented as medians and interquartile ranges—Me [Q_1_–Q_3_], while categorical variables are expressed as counts and percentages—*n* (%).

To compare quantitative parameters between independent samples, the Mann–Whitney *U* test was applied; for comparing frequencies, Pearson’s chi‐squared (*χ* ^2^) test was used. The baseline comparability of patient subgroups (those receiving vs. not receiving therapy) regarding age and disease stage was evaluated using the Mann–Whitney U and *χ* ^2^ tests, respectively. Structural analysis of respiratory impairments was conducted by ranking spirometric parameters according to the frequency of their pathological deviation from normal reference values.

Correlation analysis was performed using Spearman’s rank correlation coefficient (*r*_*s*_). Hypothesis testing was divided into two distinct blocks:

1. Exploratory analysis: during the calculation of the overall correlation matrix (comprising 11 spirometric parameters and 32 clinical symptoms; 352 tests in total), the Benjamini–Hochberg procedure (BH FDR) was applied at a threshold of *Q* = 0.05 to control the false discovery rate.
2. A priori (targeted) analysis: the evaluation of associations between respiratory parameters and bradykinesia, posture impairments, and fatigue (PFS‐16 index) was treated as a set of preliminary hypotheses and performed at the nominal significance level without corrections for multiple comparisons.

For all statistical tests, the critical level of significance was set at *p* < 0.05.

## 3. Results

The study included 57 outpatients with idiopathic PD, comprising 31 women and 26 men. The distribution of patients by disease stage according to the H&Y classification revealed a predominance of individuals with advanced motor deficits: H&Y Stage I was verified in 1 patient, Stage II in 14 patients, and Stage III in 42 patients.

At the time of the examination, dopaminergic therapy in various combinations (levodopa‐containing drugs, amantadine, and dopamine receptor agonists [DRAs]) was received by 46 (80.7%) patients, while 11 (19.3%) patients were not receiving specific antiparkinsonian treatment. None of the patients in the cohort were taking anticholinergic medications.

Impairment of QoL, assessed via the 39‐item Parkinson’s Disease Questionnaire Summary Index (PDQ‐39 SI), was registered in all examined individuals, with a median score of 30.12% (14.74–43.58) (compared to a normative value of 0%). A clinically significant syndrome of pathological fatigue, defined as a 16‐item PFS‐16 index of ≥ 3.3 scores, was identified in 43.1% of patients with PD. The comprehensive demographic and clinical characteristics of the study cohort are presented in Table 1.

**TABLE 1:** Demographic and clinical characteristics of PD patients.

Characteristics	Ме	Q1‐Q3
Age, years	62.00	57.00–70.00
PD duration, years	4.40	3.00–5.50
H&Y	3.00	2.00–3.00
Sch&En %	90.00	80.00–90.00
PDQ‐39 Summary Index, %	30.12	14.74–43.58
UPDRS Part II, scores	9.00	7.00–11.00
UPDRS Part III, scores	28.00	23.50–33.50
PFS‐16 index	3.12	1.81–4.06
Levodopa, mg/day (*n* = 30)	400.0	300.0–525.0
Amantadine, mg/day (*n* = 20)	300.0	225.0–300.0
Piribedil, mg/day (*n* = 23)	150.00	100.0–150.0
Pramipexole, mg/day (*n* = 14)	3.50	3.0–4.00

*Note:* Me, median; Q_1_–Q_3_, interquartile range (25th and 75th percentiles); *n*, number of patients receiving the specified drug. Drug dosages are presented as milligrams per day (mg/day). H&Y, Hoehn and Yahr rating scale; Sch&En, Schwab and England activities of daily living scale; PDQ‐39, 39‐item Parkinson’s Disease Questionnaire; UPDRS II, Unified Parkinson’s Disease Rating Scale, Part II (daily living); UPDRS III, Unified Parkinson’s Disease Rating Scale, Part III (motor examination); PFS‐16, 16‐item Parkinson Fatigue Scale.

Abbreviation: PD, Parkinson’s disease.

### 3.1. Comparative Analysis of Respiratory Function in PD Patients and Healthy Controls

At the initial stage of the study, a structural mapping was performed by ranking the frequencies of the detected respiratory impairments relative to standard reference values. Additionally, a comparative analysis of spirometric parameters was conducted between patients with PD and healthy individuals (the control group). The comprehensive data for all evaluated respiratory metrics in patients with PD are presented in Table 2.

**TABLE 2:** Spirographic parameters in PD patients.

Parameter	Units	Normal	Ме	Q1–Q3
VC	L		3235	2630–4002
	% pred	≥ 80%	100.00	89.35–115.07

FVC	L		2710	2230–3360
	% pred	≥ 80%	88.90	76.55–101.00

FEV_1_	L		2400	1945–3000

%pred (≥ 80%)	% pred	≥ 80%	97.30	82.35–111.55

Tiffeneau index (FEV_1_/VC)	%	≥ 70%	76.73	66.39–81.95

Gensler index (FEV_1_/FVC)	%	≥ 70%	87.60	82.27–92.22

PEF	L/s		5190	4050–7815
	% pred	≥ 80%	88.70	70.70–105.30

MMF	L/s		3180	2095–4005
	% pred	≥ 70%	114.30	81.00–139.70

FEF_25_	L/s		3960	1780–5470
	% pred	≥ 70%	90.35	62.77–115.65

FEF_50_	L/s		3650	2315–4695
	% pred	≥ 70%	103.70	71.35–132.65

FEF_75_	L/s		1.58	1.05–2.06
	% pred	≥ 70%	144.90	101.80–201.60

MVV	L		43.00	30.80–51.00
	% pred	≥ 75%	44.40	31.05–54.75

*Note:* Me, median; Q_1_–Q_3_, interquartile range (25th and 75th percentiles). Normal reference values (% pred) are based on the Knudson prediction equations (ATS standards). MMF, maximal midexpiratory flow; FEF_25_, FEF_50_, FEF_75_, forced expiratory flows at 25%, 50%, and 75% of FVC; MVV, maximum voluntary ventilation; % pred, percentage of the predicted value.

Abbreviations: FEV_1_, forced expiratory volume in 1 s; FVC, forced vital capacity; PEF, peak expiratory flow; VC, vital capacity.

An analysis of the frequency of pathological deviations from standard reference values within the PD cohort allowed for the ranking of respiratory parameters based on their vulnerability. According to the obtained data (Figure 1), the vast majority of patients (97.5%) exhibited a decrease in MVV. The second most frequent impairment was a drop in PEF, recorded in 42.1% of the patients. A reduction in parameters such as the forced expiratory flow at 25% of FVC (FEF_25_), the IT, and FVC was observed in approximately one‐third of the examined population. A decline in metrics such as FEF_50_, FEV_1_, MMF, VC, and FEF_75_ was identified somewhat less frequently. The FEV_1_G proved to be the most stable parameter, with a decrease registered in only 5.0% of cases. A visual representation of the ranking of all evaluated spirographic parameters by the frequency of their impairment in the PD group is presented in Figure 1.

**FIGURE 1:**
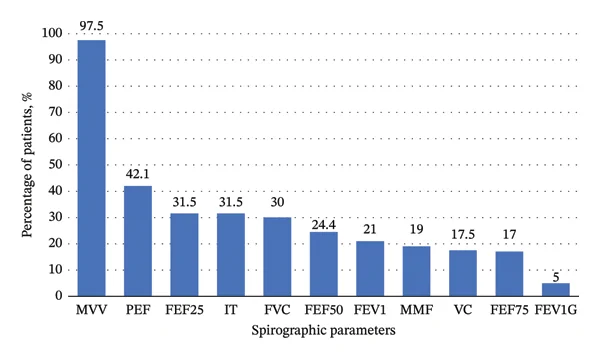
Ranking of spirographic parameters by the frequency of pathological deviations from normal reference values in the total cohort of patients with Parkinson’s disease (*n* = 57). Abbreviations: MVV: maximum voluntary ventilation; PEF: peak expiratory flow; FEF_25_, FEF_50_, FEF_75_: forced expiratory flows at 25%, 50%, and 75% of FVC; IT: Tiffeneau index; FVC: forced vital capacity; FEV_1_: forced expiratory volume in 1 s; MMF: maximal midexpiratory flow; VC: vital capacity; FEV_1_G: Gensler index.

A comparative analysis of pulmonary function parameters was conducted between the PD patient cohort (*n* = 57) and the healthy control group (*n* = 10). The groups were comparable regarding sex distribution (4 women and 6 men vs. 31 women and 26 men, respectively; *χ* ^2^ = 0.706; *p* = 0.401) and age (Me = 55.00 [53.00–62.75] and Me = 62.00 [57.00–70.00] years, respectively; *p* = 0.061). PD outpatients exhibited a statistically significant decrease in both the percentage of predicted values (VC %pred) and the absolute lung volume expressed in liters (Mann–Whitney *U* test, *p* < 0.05):

• VC %pred: Me = 100.60% (89.35–115.07) vs. Me = 118.40% (107.70–139.27) in the control group (*p* = 0.002);
• VC (L): Me = 3.24 (2.63–4.00) *L* vs. Me = 4.18 (3.43–5.30) *L* in the control group (*p* = 0.010).

No statistically significant differences between the groups were detected for the remaining evaluated spirographic parameters.

### 3.2. Evaluation of Relationships Between Respiratory Dysfunction and PD Symptoms

At a nominal significance level of *p* < 0.05, without adjustment for multiple comparisons, 81 pairwise correlations were initially identified (out of 352 tested hypotheses). However, after applying the BH procedure (FDR = 0.05) to control the false discovery rate, the number of statistically stable and significant correlations was reduced to 38. Only the pairs that successfully passed this correction threshold were subjected to further clinical and statistical analysis (Table 3).

**TABLE 3:** Spearman rank correlations between PD scale scores and spirographic parameters (absolute values).

Parameter	H&Y stage	PDQ‐39 total	PDQ‐39 emotional	PDQ‐39 cognition	UPDRS II	UPDRS III	Arising from chair	Posture	Gate	Postural stability	Body bradykinesia
VC (L)	−0,374**^∗∗^**	−0.232	−0.329	−0.333	−0,381**^∗∗^**	−0.277	−0,562**^∗∗∗^**	−0,422**^∗∗∗^**	−0,527**^∗∗∗^**	−0,444**^∗∗∗^**	−0.203
FVC (L)	−0,493**^∗∗∗^**	−0.271	−0.355	−0.314	−0,432**^∗∗^**	−0.292	−0,582**^∗∗∗^**	−0,475**^∗∗∗^**	−0,596**^∗∗∗^**	−0,464**^∗∗∗^**	−0.202
FEV_1_ (L)	−0,444**^∗∗∗^**	−0,418**^∗∗^**	−0,476**^∗∗∗^**	−0,455**^∗∗∗^**	−0.363	−0,384**^∗∗^**	−0,444**^∗∗∗^**	−0,541**^∗∗∗^**	−0,532**^∗∗∗^**	−0,548**^∗∗∗^**	−0,384**^∗∗^**
MMF (L/s)	−0.235	−0.297	−0.379	−0.319	−0.327	−0.236	−0,379**^∗∗^**	−0.30	−0.363	−0.316	−0.300
PEF (L/s)	−0.335	−0,385**^∗∗^**	−0,462**^∗∗∗^**	−0.362	−0.305	−0.240	−0,469**^∗∗∗^**	−0,396**^∗∗^**	−0,419**^∗∗∗^**	−0.355	−0.168

*Note:* r‐S, Spearman’s rank correlation coefficient. All presented correlations are statistically significant after Benjamini–Hochberg correction at FDR = 0.05 (with a total of 352 hypotheses tested in the matrix). Statistical significance levels. H&Y, Hoehn and Yahr stage; PDQ‐39, Parkinson’s Disease Questionnaire; UPDRS II, Unified Parkinson’s Disease Rating Scale Part II; UPDRS III, Unified Parkinson’s Disease Rating Scale Part III; MMF, maximal midexpiratory flow.

Abbreviations: FEV_1_, forced expiratory volume in 1 s; FVC, forced vital capacity; PD, Parkinson’s disease; PEF, peak expiratory flow; VC, vital capacity.

^∗∗^ *p* < 0.01.

^∗∗∗^ *p* < 0.001.

Association of Disease Severity (H&Y Stage) with Respiratory Function (Table 3). The H&Y stage demonstrated highly significant inverse correlations with both lung volumes and expiratory flow parameters: VC (*r*_*s*_ = −0.374; *p* = 0.005), FVC (*r*_*s*_ = −0.493; *p* < 0.0001), and FEV_1_ (*r*_*s*_ = −0.444; *p* = 0.0005).

This pattern was prominently reflected in the subgroup comparative analysis: patients with H&Y Stage III PD exhibited a statistically significant decrease in FVC %pred compared to those with Stage II disease (Me = 85.20% [70.80–97.70] vs. Me = 95.00% [88.70–103.00], respectively; *p* = 0.033 by the Mann–Whitney *U* test).

Categorical frequency analysis confirmed that progression to a more advanced disease stage is coupled with the accumulation of restrictive ventilatory defects (Pearson’s *χ* ^2^ test). A drop in the FVC %pred parameter below normal reference thresholds occurred significantly more often in Stage III than in Stage II patients—38.1% vs. 7.1% of cases, respectively (16 out of 42 patients vs. 1 out of 14 patients; *χ* ^2^ = 4.758, *p* = 0.030).

These results may indicate a clear trend toward worsening lung ventilatory capacity (primarily driven by the restrictive component) in line with general disease progression and increasing clinical stages of PD.

Relationship of Respiratory Function with Patients’ Activities of Daily Living and Quality of Life (Table 3). An analysis of patients’ activities of daily living (UPDRS Part II) revealed significant inverse correlations with static lung volumes: VC (*r*_*s*_ = −0.381; *p* = 0.004) and FVC (*r*_*s*_ = −0.432; *p* = 0.001). When evaluating the patients’ QoL using the integral PDQ‐39 SI, reliable inverse correlations were found with expiratory flow parameters: FEV_1_ (*r*_*s*_ = −0.418; *p* = 0.002) and PEF (*r*_*s*_ = −0.385; *p* = 0.005).

An analysis of individual PDQ‐39 subscales demonstrated that psychoemotional and cognitive impairments made the most pronounced contribution to the structure of these relationships. Specifically, the level of emotional well‐being was highly significantly correlated with FEV_1_ (*r*_*s*_ = −0.476; *p* < 0.001) and PEF (*r*_*s*_ = −0.462; *p* < 0.001). Furthermore, the severity of cognitive problems demonstrated a stable inverse association with the FEV_1_ parameter (*r*_*s*_ = −0.455; *p* < 0.001).

Thus, the decline in lung ventilatory capacity in PD is associated not only with increasing motor deficits but is also coupled with an objective limitation of patients’ daily independence, a reduction in their overall QoL, and a compromise of their emotional and cognitive well‐being.

Relationship of Respiratory Function with Patients’ Motor Symptoms (Table 3). An analysis of the overall severity of motor deficits showed that the total score on the UPDRS Part III motor scale was significantly inversely correlated with FEV_1_ (*r*_*s*_ = −0.384; *p* = 0.003).

The investigation of individual motor symptoms revealed heterogeneity in their relationship with lung ventilatory function. Specifically, the entire cluster of axial and locomotor impairments, which includes disorders of posture, gait, postural stability, and difficulty arising from a chair, stably inversely correlated with both static volumes (VC and FVC) and all expiratory flow parameters (FEV_1_, MMF, and PEF). At the same time, body bradykinesia demonstrated an isolated inverse association exclusively with the FEV_1_ parameter. Notably, no statistically significant correlations were found between spirographic metrics and the severity of tremor or rigidity in the examined patient cohort.

Given the primary role of axial deficits, bradykinesia, and key nonmotor features in compromising patients’ QoL, a detailed multilevel analysis was subsequently performed to explore the associations of posture impairments, bradykinesia, and fatigue syndrome with the patients’ respiratory function.

### 3.3. Posture

During the screening analysis of the overall dataset, the severity of posture impairments was found to significantly correlate with key volumetric and expiratory flow parameters of pulmonary function: VC, FVC, FEV_1_, and PEF.

To perform a detailed evaluation of the relationship between axial impairments and respiratory metrics, a comparative analysis of contrasting patient subgroups was conducted. Patients with moderate‐to‐severe posture impairments (Scores 2–3 on UPDRS Part III, *n* = 23) demonstrated a statistically significant decrease in most expiratory flow parameters compared to those without pronounced postural disorders (Scores 0–1 on UPDRS Part III, *n* = 34; Mann–Whitney *U* test):

• FEV_1_%pred: Me = 86.50% (77.30–100.00) vs. Me = 102.50% (88.27–112.95) (*p* = 0.037);
• PEF %pred: Me = 73.80% (45.10–94.60) vs. Me = 98.95% (77.60–110.87) (*p* = 0.005);
• FEF_25_%pred: Me = 71.80% (41.65–95.40) vs. Me = 96.10% (74.60–120.70) (*p* = 0.022);
• IT: Me = 71.42% (62.28–77.61) vs. Me = 78.80% (70.24–83.81) (*p* = 0.017).

Categorical frequency analysis confirmed that posture impairment is coupled with a drop in pulmonary ventilatory capacity below normal reference values. Specifically, in the subgroup of patients with moderate‐to‐severe posture impairments (Scores 2–3), deviations from the norm occurred significantly more frequently for the following parameters (Pearson’s *χ* ^2^ test):

• PEF %pred: deviations were identified in 65.2% of patients vs. 26.5% in the comparison group (15 out of 23 vs. 9 out of 34 individuals; *χ* ^2^ = 8.449, *p* = 0.004);
• FEF_25_%pred: in 42.9% vs. 17.6% of patients, respectively (9 out of 21 vs. 6 out of 34 individuals; *χ* ^2^ = 3.895, *p* = 0.049);
• IT: in 47.8% vs. 21.2% of patients, respectively (11 out of 23 vs. 7 out of 33 individuals; *χ* ^2^ = 4.401, *p* = 0.036).

A visual representation of the distribution of pathological spirographic deviations across the evaluated patient groups is presented in Figure 2.

**FIGURE 2:**
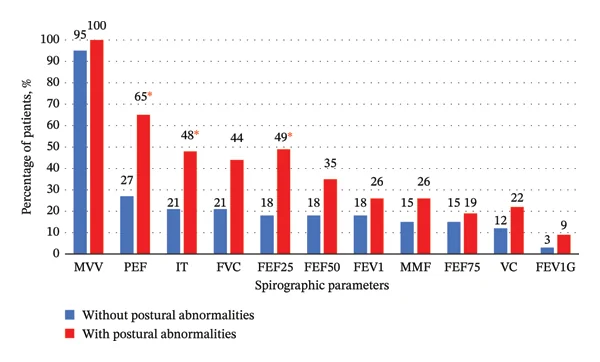
Frequencies of reduced spirographic parameters (% pred) in PD patients with and without pronounced postural abnormalities. Blue bars: patients without pronounced postural abnormalities (Scores 0–1 on MDS‐UPDRS Part III, *n* = 34); red bars: patients with moderate‐to‐severe postural abnormalities (Scores 2–3 on MDS‐UPDRS Part III, *n* = 23). ^∗^Statistically significant differences between the groups (*p* < 0.05 by Pearson’s *χ* ^2^ test). Abbreviations: MVV: maximum voluntary ventilation; PEF: peak expiratory flow; IT: Tiffeneau index; FVC: forced vital capacity; FEF_25_, FEF_50_, FEF_75_: forced expiratory flows at 25%, 50%, and 75% of FVC; FEV_1_: forced expiratory volume in 1 s; MMF: maximal midexpiratory flow; VC: vital capacity; FEV_1_G: Gensler index.

Thus, the obtained results indicate that the progression of postural abnormalities in PD is closely coupled with the development of combined ventilatory defects, significantly limiting both the expiratory flow reserves of forced expiration and overall airway patency.

### 3.4. Body Bradykinesia

This parameter demonstrated a selective pattern of associations aimed specifically at the expiratory flow component of the respiratory act. During the screening analysis, a direct inverse correlation was identified between the severity of bradykinesia and the FEV_1_.

To verify the detected association, the patients were divided into subgroups based on the severity of this motor deficit. In patients with pronounced bradykinesia (scores > 1 on the UPDRS Part III scale, *n* = 28), a statistically significant decrease in key expiratory parameters was recorded compared to those with mild manifestations (Score 1, *n* = 29; Mann–Whitney *U* test):

• FEV_1_%pred: Me = 86.60% (75.35–106.25) vs. Me = 103.00% (88.80–113.55) (*p* = 0.017);
• IT: Me = 71.42% (62.28–79.20) vs. Me = 77.94% (70.64–83.46) (*p* = 0.048);
• FEV_1_G: Me = 85.00% (78.30–89.56) vs. Me = 89.00% (83.66–94.99) (*p* = 0.039).

Additionally, categorical frequency analysis demonstrated that the worsening of bradykinesia is coupled with an increase in the proportion of patients exhibiting a drop in expiratory flow reserves below normal reference thresholds. In the subgroup with pronounced bradykinesia, the frequency of deviations from the norm for the MMF %pred parameter was significantly higher, being registered in 32.1% of patients vs. 6.9% in the subgroup with mild bradykinesia (9 out of 28 vs. 2 out of 29 individuals, respectively; *χ* ^2^ = 5.221, *p* = 0.023).

Thus, the comprehensive analysis supports the initial exploratory hypothesis: the worsening of bradykinesia in line with PD progression is coupled with a decline in the expiratory effort of the respiratory musculature, manifesting as the development and exacerbation of obstructive ventilatory defects (reflected by the decline in FEV_1_, IT, FEV_1_G, and MMF).

### 3.5. Fatigue

Of particular interest was the targeted analysis of the relationships between pulmonary function and the severity of nonmotor manifestations of PD, specifically the fatigue syndrome evaluated by the PFS‐16 scale. The study revealed a stable pathogenetic triad linking pathological fatigue to a decline in expiratory flow parameters.

When analyzing continuous variables, the integral PFS‐16 fatigue index was significantly inversely correlated with the flow parameters of forced expiration: FEV_1_ (*r*_*s*_ = −0.311; *p* = 0.026) and PEF (*r*_*s*_ = −0.366; *p* = 0.008). It is worth noting that these correlations were at the nominal significance level (*p* < 0.05); however, given the comprehensive nature of testing this a priori hypothesis, they were thoroughly analyzed in the subsequent stages.

To verify the detected trend, patients were divided into two subgroups depending on the severity of their symptoms (pathological fatigue index ≥ 3.3 vs. normal index). The subgroup comparison fully supported the results of the correlation analysis: Patients with pronounced fatigue exhibited a statistically significant decrease in the percentage of predicted PEF (PEF %pred) compared to individuals with normal fatigue levels (Me = 72.80% [51.82–97.45] vs. Me = 97.80% [76.45–113.95], *p* = 0.025 by the Mann–Whitney *U* test).

Finally, categorical frequency analysis demonstrated that a drop in the PEF %pred parameter below normal reference thresholds occurred significantly more often in individuals with fatigue syndrome (PFS index ≥ 3.3) than in the comparison group—63.6% vs. 27.6% of cases, respectively (14 out of 22 patients vs. 8 out of 29 patients; *χ* ^2^ = 6.628, *p* = 0.011).

Thus, the combined application of correlation, subgroup comparative, and frequency analyses reliably verifies the association of expiratory flow limitation and decreased expiratory flow reserves (PEF, FEV_1_) with the severity of subjective fatigue in PD, indicating an important respiratory component within the structure of this nonmotor syndrome.

### 3.6. Evaluation of Associations Between Antiparkinsonian Therapy and Pulmonary Function Parameters

The statistical evaluation was performed both regarding the fact of receiving specific treatment in general and in relation to the daily dosages of individual drug classes.

To explore the relationship with dopaminergic therapy in general, a subgroup comparison was conducted between 46 patients receiving specific treatment (levodopa‐containing drugs, DRAs, and amantadine) and 11 patients who were untreated at the time of the examination. The evaluated subgroups were fully comparable regarding age (*p* = 0.352) and H&Y stage (*p* = 0.484).

The exploratory comparative analysis revealed that the untreated patients exhibited a statistically significant and clinically pronounced decline in both volumetric and expiratory flow parameters compared to those receiving therapy (Mann–Whitney *U* test):

• FVC %pred: Me = 83.10% (68.70–88.70) vs. Me = 91.30% (77.85–104.15) (*p* = 0.039);
• FEV_1_%pred: Me = 82.80% (68.00–96.00) vs. Me = 101.00% (86.32–112.55), respectively (*p* = 0.015);
• FEF_75_%pred: Me = 105.55% (67.17–144.42) vs. Me = 153.40% (105.50–233.30), respectively (*p* = 0.039);
• IT: Me = 67.60% (58.88–79.55) vs. Me = 77.55% (69.02–83.10), respectively (*p* = 0.042).

A visual representation of the structure of respiratory impairments associated with treatment status is presented in Figure 3, clearly illustrating that the frequency of reduced spirometric parameters was higher in the untreated subgroup across most variables.

**FIGURE 3:**
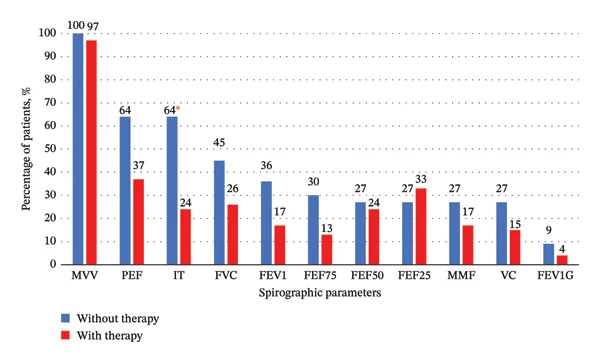
Frequency of pathological reductions in spirometric parameters in patients with Parkinson’s disease with and without dopaminergic therapy. Blue bars represent patients without antiparkinsonian therapy (*n* = 11); red bars represent patients receiving specific dopaminergic treatment (*n* = 46). ^∗^Statistically significant differences between the groups (*p* < 0.05 by Pearson’s *χ* ^2^ test). Abbreviations: MVV: maximum voluntary ventilation; PEF: peak expiratory flow; IT: Tiffeneau index; FVC: forced vital capacity; FEV_1_: forced expiratory volume in 1 s; FEF_25_, FEF_50_, FEF_75_: forced expiratory flows at 25%, 50%, and 75% of FVC; MMF: maximal midexpiratory flow; VC: vital capacity; FEV_1_G: Gensler index.

Categorical analysis of fourfold contingency tables indicated that a pathological drop in key respiratory parameters below normal reference thresholds occurred more frequently in patients without therapy than in those receiving treatment.

The statistically significant differences and prominent clinical trends were distributed as follows:

• IT: a drop below the normal threshold (obstructive pattern) was detected in 63.64% of patients without treatment versus only 23.91% in the treated group (7 out of 11 vs. 11 out of 46 individuals; Pearson’s *χ* ^2^ = 6.483, *p* = 0.011), proving a statistically robust protective effect of dopaminergic therapy;
• PEF %pred: a reduction below the normal threshold was registered in 63.64% of patients without therapy versus 36.96% of those receiving treatment (7 out of 11 vs. 17 out of 46 individuals; *p* = 0.161 via Fisher’s exact test), demonstrating a clear clinical trend toward preservation of integral expiratory power under treatment conditions;
• FEV_1_%pred: a pathological decrease was observed in 36.36% of individuals without treatment and dropped to 17.39% in the therapy group (4 out of 11 vs. 8 out of 46 patients; *p* = 0.223 via Fisher’s exact test), reflecting a consistent clinical trend toward minimizing obstructive deficit under the influence of specific antiparkinsonian medications.

For the remaining spirometric volume and flow parameters, no statistically significant differences or pronounced clinical trends were observed between the study groups.

Further analysis focused on exploring the dose‐dependent relationships of specific classes of antiparkinsonian drugs. The identified associations successfully passed the multiple comparisons correction procedure using the BH method (FDR = 0.05) and demonstrated a high selectivity of action.

During the analysis, significant direct correlation links were verified between the daily dosage of amantadine‐group medications and a wide spectrum of both volumetric and expiratory flow parameters of pulmonary function: VC %pred (*r*_*s*_ = 0.467; *p* = 0.0003), FVC %pred (*r*_*s*_ = 0.518; *p* < 0.0001), FEV_1_%pred (*r*_*s*_ = 0.467; *p* = 0.0002), MMF %pred (*r*_*s*_ = 0.422; *p* = 0.0011), FEF_75_%pred (*r*_*s*_ = 0.515; *p* = 0.001), PEF%pred (*r*_*s*_ = 0.578; *p* < 0.0001), and MVV %pred (*r*_*s*_ = 0.461; *p* = 0.002).

Similarly, the daily dose of DRAs demonstrated direct relationships; however, their character was more selective, primarily affecting the expiratory flow characteristics of forced expiration and parameters of airway patency. Reliable positive correlations for the DRA dose were registered for the FEV_1_G (FEV_1_/FVC) (*r*_*s*_ = 0.432; *p* = 0.001), as well as the MMF %pred (*r*_*s*_ = 0.411; *p* = 0.001) and FEF_75_%pred (*r*_*s*_ = 0.498; *p* = 0.001) metrics.

Notably, no statistically significant associations were found between the daily dosage of levodopa‐containing drugs and any of the evaluated spirographic indicators in our patient cohort.

Thus, the lack of specific antiparkinsonian therapy is associated with worse indicators of lung ventilatory function, which may indicate a potential positive dose‐dependent trend of amantadine and DRAs regarding respiratory status. The multiple relationships of respiratory metrics with amantadine and DRAs, in the absence of associations with levodopa, may suggest that the optimization of respiratory status in PD may be largely mediated by nondopaminergic (specifically, glutamatergic) mechanisms, as well as by the specific modulating action of DRAs.

## 4. Discussion

The data obtained in this study indicate a high prevalence of respiratory dysfunction in outpatients with PD at H&Y Stages II–III of the disease. When comparing the spirographic parameters with the healthy control group, we, consistent with a number of authors [7, 9, 11], identified a statistically significant decrease in the mean VC values in the PD cohort, suggesting a predisposition to restrictive ventilatory defects.

Of all the spirographic parameters evaluated in this study (Figure 1), a decrease in MVV relative to predicted values was registered most frequently, occurring in 97.5% of the patients. These data are consistent with the findings of Polatli M. et al. [15], who concluded that the MVV parameter is the most severely affected lung function metric in PD. It is generally accepted that this index accurately reflects the activity of the respiratory musculature, the contraction frequency and force of which may be compromised by the presence of bradykinesia, rigidity, and postural disorders. Aside from MVV, less coordinated and delayed muscle function is apparently associated with a reduction in other spirometric flow rate parameters. Specifically, a drop in PEF was observed in 42.1% of our patients, while approximately one‐third of the cohort exhibited a decrease in FEF_25_ and IT metrics, pointing to the involvement of an obstructive component within the structure of respiratory dysfunction.

A decrease in FVC and VC parameters, characteristic of restrictive ventilatory defects, was noted in 30.0% and 17.5% of the patients, respectively (Figure 1). It is hypothesized that the development of a restrictive pattern of respiratory dysfunction in PD is also linked to abnormal muscle activity, namely, bradykinesia, rigidity, and, to some extent, tremor, which interferes with the coordinated function of the respiratory muscles. Furthermore, postural disorders (posture impairments and progressive arthrosis) appear to contribute to this process. The combination of these factors reduces the elasticity and mobility of the chest wall, increasing the rigidity of its biomechanical framework, and is associated with muscle weakness, thereby limiting tidal volume and inspiratory flow.

Thus, patients with PD exhibit plausible pathogenetic prerequisites for the coexistence of both obstructive and restrictive components of respiratory dysfunction, the severity of which demonstrates a trend toward exacerbation in line with the worsening of motor symptoms [9, 34].

### 4.1. Respiratory Dysfunction in the Context of Disease Progression, Activities of Daily Living, and QoL

The severity of respiratory dysfunction in the examined patients was closely coupled with the disease stage according to the H&Y scale across a number of key pulmonary function parameters, including VC, FVC, and FEV_1_ (Table 3). Concurrently, a decrease in the FVC %pred parameter was registered significantly more frequently in patients with H&Y Stage III PD than in those with Stage II disease. On the other hand, no statistically significant relationships were found between respiratory impairments and PD duration in our cohort, although such a dependency has been described by several other investigators [4, 9, 35].

The patients’ activities of daily living, evaluated using the Sch&En scale and the UPDRS Part II subscale, were closely interrelated with expiratory flow parameters, as well as with VC and FVC values. The negative associations of respiratory parameters with the QoL of our examined patients were definitely detrimental, despite the predominantly mild‐to‐moderate severity of their breathing disorders, which is consistent with the results of previous studies [1, 2].

For instance, in a study by Santos R.B.D. et al., the overall QoL score via the PDQ‐39 questionnaire and the mobility domain score inversely correlated with VC, FEV_1_, and PEF parameters [9]. In our work, the overall PDQ‐39 scores, as well as the emotional well‐being and cognition domains (Table 3), were coupled predominantly with expiratory flow parameters (FEV_1_ and PEF).

Thus, the obtained results point to a potential negative impact of respiratory dysfunction on both activities of daily living and the overall QoL in patients with PD, emphasizing the importance of timely diagnosis for not only motor but also functional pulmonary disorders.

### 4.2. Impact of Motor Symptoms and Postural Impairments on the Development of Respiratory Dysfunction

The relationship between individual motor symptoms of PD and respiratory dysfunction was analyzed by comparing the MDS‐UPDRS Part III scores with spirographic parameters (Table 3). It was established that the worsening of motor deficits is coupled with a decline in lung ventilatory function: A statistically significant inverse correlation was found between the total UPDRS Part III score and the forced FEV_1_. Concurrently, the maximum number of associations was recorded between the worsening of postural‐locomotor impairments (difficulty arising from a chair, alterations in posture, gait, and postural stability) and a decrease in both expiratory flow parameters, as well as VC and FVC metrics, pointing to the coexistence of both obstructive and restrictive components of the respiratory deficit.

It is generally accepted that the respiratory system in PD patients is vulnerable to the progression of overall motor deficits; concurrently, the restriction of chest wall expansion is closely interrelated with posture impairments and osteoarticular degeneration, the combined impact of which is hypothesized to alter the typical mechanics of the respiratory act [11]. The significant inverse correlations found in our study between the posture score on the UPDRS Part III scale and the VC, FVC, FEV_1_, and PEF metrics (Table 3) are consistent with this pathogenetic concept.

Further support for this interrelation is provided by the results of the comparative analysis of contrasting patient subgroups: Individuals with pronounced posture impairments exhibited significantly lower values and more frequent pathological deviations in expiratory flow rates and the IT (Figure 2). Thus, pulmonary function parameters in the examined cohort demonstrate a close association with the severity of postural disorders.

In the course of the study, an inverse correlation was also verified between a key motor symptom of PD—bradykinesia—and the FEV_1_ parameter (Table 3). In the subgroup comparative analysis, patients with more pronounced manifestations of bradykinesia exhibited significantly lower values for FEV_1_, IT, and FEV_1_G metrics. These results are partially consistent with the findings of previous studies, which demonstrated that an increase in motor deficits, including bradykinesia and rigidity, is associated with a decline in pulmonary function and altered mechanics of the respiratory act [9, 35, 36]. At the same time, no convincing data supporting an association between spirographic parameters and the severity of rigidity or tremor were obtained in our patient cohort.

Relying on the results of our analysis, it can be suggested that among the core motor manifestations of PD, it is the postural‐locomotor impairments (disorders of posture, gait, and stability) that demonstrate the closest association with the development of respiratory dysfunction. These impairments are linked to key spirometric parameters reflecting both the restrictive and obstructive components of breathing disorders. Postural abnormalities are most characteristic of advanced stages of the disease, starting from H&Y Stage III, which may pathogenetically explain the worsening of pulmonary function metrics observed in these patients compared to those with Stage II PD.

In turn, bradykinesia in the examined cohort was interrelated predominantly with expiratory flow parameters, which may indicate its selective contribution primarily to the formation of the obstructive component of the respiratory deficit.

### 4.3. Impact of Respiratory Dysfunction on Pathological Fatigue Syndrome

Fatigue syndrome represents another significant clinical phenomenon demonstrating a close association with the development of respiratory dysfunction in PD. According to literature data, pathological fatigue is an extremely common and practically pathognomonic manifestation of PD, registered in 33%–82% of patients; however, the exact mechanisms underlying its development remain controversial to date [37, 38].

In our examined cohort, a clinically pronounced fatigue syndrome via the PFS‐16 scale was identified in 43.1% of patients, which completely aligns with global epidemiological trends. Concurrently, the PFS‐16 score significantly correlated with expiratory flow parameters—FEV_1_ and PEF.

Further support for this interrelation is provided by the results of the subgroup comparative analysis: In individuals with PD and an elevated fatigue index, a drop in PEF was registered significantly more frequently and was more pronounced than in patients with normal fatigue levels.

The obtained results are consistent with the modern concept of the multifactorial nature of nonmotor manifestations in PD and point to a potential pathogenetic link between chronic respiratory deficits (specifically, expiratory flow impairment) and the subjective perception of fatigue in this category of patients.

## 5. Impact of Dopaminergic Pharmacotherapy on Respiratory Dysfunction

The impact of dopaminergic therapy on the severity of respiratory dysfunction in PD remains controversial in the current literature [5, 22, 38–40]. In the course of the exploratory analysis of our cohort, it was established that patients receiving dopaminergic therapy (*n* = 46) exhibited significantly higher values of quantitative spirometric parameters, including FVC %pred, FEV_1_%pred, and the IT, compared to untreated individuals (*n* = 11). Furthermore, a categorical frequency analysis demonstrated that a pathological drop below normal thresholds for the IT was registered significantly more frequently in the untreated subgroup (Figure 3), while prominent clinical trends toward a higher prevalence of respiratory deficit in untreated patients were observed for PEF %pred and FEV_1_%pred values. Collectively, these data indicate a potential positive impact of dopaminergic treatment primarily on the obstructive components of the respiratory deficit, as well as on the preservation of integral expiratory volume and velocity reserves.

An analysis of the dose‐dependent effects of specific pharmacological classes revealed a high selectivity in their relationship with respiratory function. Significant direct (positive) correlation links were verified between the daily dosage of amantadine‐group medications and a wide spectrum of both volumetric and expiratory flow parameters of pulmonary function (VC %pred, FVC%pred, FEV_1_%pred, MMF%pred, FEF_75_%pred, PEF%pred, and MVV%pred). Concurrently, the daily dose of DRAs demonstrated direct relationships exclusively with the flow rate characteristics of forced expiration and parameters of airway patency (FEV_1_G, MMF%pred, and FEF_75_%pred). Notably, no statistically significant associations were found between the daily dosage of levodopa‐containing drugs and any of the evaluated spirographic indicators in our patient cohort.

It is generally accepted that all core motor symptoms are involved in the development of respiratory disorders in PD [8, 9, 34]; consequently, it is logical that dopaminergic therapy, by reducing the severity of motor deficits, is associated to some extent with more preserved parameters of pulmonary function [35]. The fact that higher daily doses of amantadine and DRAs are coupled with a more preserved respiratory status, while an equivalent relationship is absent for levodopa, suggests the potential involvement of nondopaminergic mechanisms in respiratory regulation in PD. The blockade of NMDA glutamate receptors by amantadine may exert a central protective effect on the brainstem respiratory centers, overcoming glutamatergic excitotoxicity [41], whereas DRAs may gently modulate the tone of the bronchial smooth muscle via a specific receptor apparatus [42, 43].

Nevertheless, according to the obtained findings, antiparkinsonian pharmacotherapy is not capable of completely eliminating the problem of respiratory dysfunction, which dictates the necessity of developing comprehensive, interdisciplinary approaches to the rehabilitation of these patients.

The systemic decline in lung ventilatory capacity and expiratory flow parameters identified in our cohort carries significant clinical implications. In the long term, the progression of respiratory dysfunction in PD may eventually lead to an exacerbation of dyspnea and sharply increase the risk of aspiration pneumonia, which remains one of the leading causes of mortality in this category of patients. Our data regarding the close relationship of the respiratory deficit with posture, bradykinesia, and fatigue substantiate the urgent need for implementing physical rehabilitation methods. While the current work focused on the exploratory analysis of the structure of these impairments, the development and clinical verification of specific therapeutic methods (including specialized breathing exercises and targeted physical training protocols) warrant subsequent, independent prospective investigation [4, 44, 45].

### 5.1. Study Limitations

The presented study has several methodological limitations that should be acknowledged:

1. Cross‐Sectional Design: The one‐time, cross‐sectional nature of the study allows for the identification of statistical associations only and strictly precludes the establishment of direct causal relationships.
2. Sample Imbalance: The relatively small control group (*n* = 10) and the pronounced asymmetry across the H&Y disease stages (with a predominance of Stage III, *n* = 42; and only 1 patient in Stage I) reduce the statistical power of subgroup comparisons and limit the generalizability of the findings to the early stages of PD.
3. Selection Criteria: The strict exclusion of patients with moderate‐to‐severe dementia limits the relevance of the obtained findings exclusively to cognitively intact individuals or those with mild cognitive impairment.
4. Exploratory Analysis of Therapy: The comparative evaluation between treated and untreated patients is exploratory in nature, as the observational design remains vulnerable to unmeasured confounding and treatment selection bias.

To achieve definitive verification of the pathogenetic role of motor symptoms and prospective therapeutic effects, subsequent longitudinal prospective studies on larger, well‐balanced cohorts utilizing advanced inspiratory flow profiling are warranted.

## 6. Conclusion

This study demonstrates a high prevalence of respiratory dysfunction in outpatients with H&Y Stage II–III Parkinson’s disease, revealing its close association with axial‐locomotor impairments, bradykinesia, and pathological fatigue. It was established that the ventilatory deficit significantly limits the patients’ activities of daily living and reduces their overall QoL. The obtained exploratory data suggest a potential compensating role of antiparkinsonian pharmacotherapy (specifically, amantadine and DRAs), substantiating the clinical need for implementing routine spirometric monitoring and multidisciplinary respiratory rehabilitation programs into the management of patients with PD.

## Author Contributions

All authors contributed to the study conception and design. A. A. Pilipovich performed clinical and neurological examinations and data collection. A. A. Pilipovich and S. A. Makarov performed the statistical analysis. A. A. Pilipovich, O. V. Vorob’eva, and N. N. Shindryaeva drafted the manuscript.

## Funding

The authors received no financial support from any organization for the research, authorship, and/or publication of this article.

## Disclosure

All authors read and approved the final manuscript.

## Conflicts of Interest

The authors declare no conflicts of interest.

## Data Availability

The clinical and spirometric datasets analyzed during the current study are available from the corresponding author upon reasonable request.
